# Editorial: Methods and applications in addiction psychiatry research: 2021

**DOI:** 10.3389/fpsyt.2022.1063269

**Published:** 2022-12-14

**Authors:** Marco Colizzi, Danilo De Gregorio

**Affiliations:** ^1^Unit of Psychiatry, Department of Medicine (DAME), University of Udine, Udine, Italy; ^2^Division of Neuroscience, San Raffaele Scientific Institute, Vita-Salute San Raffaele University, Milan, Italy

**Keywords:** addiction psychiatry, cannabis use disorder, alcohol use disorder, substance use disorder, gambling, Benzodiazepin, nicotine addiction, virtual reality

Addiction psychiatry research is currently in a continuous developmental stage and it is a field in medicine where the diseases to be studied keep growing in number. Nicotine addiction, cannabis use disorder (CUD), online gambling, or alcohol use disorder are a few examples (1).

In this Research Topic, we highlighted some of the current challenges and advances in this field. We tried and approached the entire landscape by underscoring the point that progress is being made in different aspects to help us deal with addictions better as a system. We included insights about novel and promising approach.

One of the points emerged in the Research Topic was the treatment of the nicotine addiction.

Historically, the treatment of nicotine addiction has been given a low priority in the overall field of addiction, compared with other substance use disorders. Both genetic and environmental are the risk factors for the smoking and subsequent tobacco addiction and many smokers are ambivalent about quitting smoking (2). The systematic review by Vanderkam et al. analyzes the duration of the efficacy of electronic cigarettes containing nicotine on smoking cessation. They found highlighting that abstinence at the end of the intervention was higher in the nicotine electronic cigarette group, compared to nicotine replacement therapy. Different study protocols have also been proposed. Indeed, Hawes et al. aimed at increasing nicotine replacement therapy adherence and smoking cessation among groups of individuals with high smoking rates and low rates of pharmacotherapy use such as those involved in the criminal legal system. Zamboni, Campagnari et al. investigate the potential role of the safe and low-cost drug cytisine to treat nicotine addiction. They compare the effects of combining cytisine with nirdosh, a herbal tobacco substitute, to cytisine only in two groups of patients also undergoing exposure to different virtual reality (VR) settings.

For instance, VR is experiencing an increased used for the assessment, diagnosis and treatment of mental diseases. VR is a computer-generated simulation of the three-dimensional environment with which it is possible to interact in a ostensibly real way (3). In this regard, Wen et al. propose the protocol for a randomized, double-blind, parallel group trial to investigate the effectiveness of transcranial magnetic stimulation (TMS) treatment after VR retrieval in reducing self-reported craving of metamphetamine and drug-related cue reactivity. A VR study protocol is proposed by Giordano et al. too, where they assess the ability of a psychological treatment to treat gambling disorder based on the combination and integration of VR cue exposure therapy and traditional cognitive behavioral therapy, thus proposing a virtual game designed for purposes other than entertainment.

Another point highlighted in the Research Topic is the treatment of CUD which is defined as the impotence to stop consuming cannabis even when it is causing physical or psychological harm. Currently, no effective pharmacological approaches for CUD are available (4). A non-randomized, open-label pilot study conducted by Cleirec et al. assesses the potential of cannabidiol [CBD, the main non-addictive cannabis compound (5)] inhaled using a vaping device in CUD. At the end of the follow-up, 6 users reduce the cannabis consumption by at least 50%., It also demonstrates that people with CUD can use an electronic cigarette as a tool to reduce their cannabis use. Moreover, Ramaekers et al. propose a perspective article highlighting the effect of acute Δ9-tetrahydrocannabinol (THC, the main psychotropic compound of cannabis) in neuroadaptative changes in chronic cannabis users. Their approach includes the quantification of neurochemical and functional brain network alterations in response to an acute THC administration. A randomized, double-blind clinical study conducted by Theunissen et al. attempts to compare the psychoactive effects of natural cannabis (THC) and JWH-018, a synthetic cannabinoid exerting psychotropic effects. The authors find that both drugs impair psychomotor and divided attention with no significant differences between the two drugs. These effects are coupled to psychotomimetic effects, even though dissociative effects are higher for JWH-018, compared to THC.

Benzodiazepines (BDZ) are the first-choice drugs used for the treatment of sleep disturbances and, in some cases, anxiety disorders (6). Zamboni, Portoghese et al. conduct a study to evaluate dependence to high BDZ doses in an Italian sample of 1,354 participants in which they investigate to which extent participants use also other substances such as tobacco, cannabis, alcohol, cocaine and heroin. The authors perform class analysis to identify the use patterns of these drugs. They divided the participants in 3 classes, finding that: participants in class 1 are mostly characterized by young men with the highest probability of using cocaine and alcohol; participants of class 2 are mostly subjects with the highest probability of being former cocaine, THC, heroin and alcohol users; the class 3 is mostly represented by women BZD users.

It has been demonstrated that chronic alcohol produce o cognitive impairements (7). Si et al. overviews how an anti-saccade task can be used as a tool to study the cognitive dysfunctions related to alcohol consumption. Also, authors propose this anti-saccade task for the early detection of relapsing risk of alcohol dependence.

The past decades have experienced the interest in the etiology of substance use disorder and addiction. Several theories highlight that addiction depends not only on alterations in neurobiological and psychological reward mechanisms, but also on the effort to cope with negative emotional experiences. In this context, Feingold and Bitain propose a clinical approach which addresses these aspects to discuss benefits for clinicians and patients working with and through addiction. Moreover, a research article proposed by Santos de Pascual et al. aims to investigate the efficacy of a multimodal treatment for a certain addict population. Their data indicate that changes occur in individuals with drug use during treatment which is also correlated to the complex social reality which is the cause of suffering to people and their relatives.

In conclusion, aware that we are still skimming just the surface of very complex phenomena, further research directions in addiction psychiatry must be specifically implemented. We need to try and better investigate the preventive potential of tools aiming at controlling and limiting substance use disorders. Finally, specific measures must be developed toward new and non-substance-related addictions whose needs are still largely unmet.

## Author contributions

All authors listed have made a substantial, direct, and intellectual contribution to the work and approved it for publication.
